# Herpesviruses in the Activated Phosphatidylinositol-3-Kinase-δ Syndrome

**DOI:** 10.3389/fimmu.2018.00237

**Published:** 2018-02-23

**Authors:** Jeffrey I. Cohen

**Affiliations:** ^1^Medical Virology Section, Laboratory of Infectious Diseases, National Institute of Allergy and Infectious Diseases, National Institutes of Health, Bethesda, MD, United States

**Keywords:** phosphatidylinositol-3-kinase, Akt, PIK3CD, PIK3R1, APDS, PASLI, Epstein–Barr virus, cytomegalovirus

## Abstract

The phosphatidylinositol-3-kinase (PI3K)/Akt pathway is important for multiple stages of herpesvirus replication including virus entry, replication, latency, and reactivation. Recently, patients with gain-of-function mutations in the p110δ-catalytic subunit of PI3K or in the p85-regulatory subunit of PI3K have been reported. These patients have constitutively active PI3K with hyperactivation of Akt. They present with lymphoproliferation and often have infections, particularly recurrent respiratory infections and/or severe virus infections. The most frequent virus infections are due to Epstein–Barr virus (EBV) and cytomegalovirus (CMV); patients often present with persistent EBV and/or CMV viremia, EBV lymphoproliferative disease, or CMV lymphadenitis. No patients have been reported with CMV pneumonia, colitis, or retinitis. Other herpesvirus infections have included herpes simplex pneumonia, recurrent zoster, and varicella after vaccination with the varicella vaccine. Additional viral infections have included adenovirus viremia, severe warts, and extensive Molluscum contagiosum virus infection. The increased susceptibility to virus infections in these patients is likely due to a reduced number of long-lived memory CD8 T cells and an increased number of terminally differentiated effector CD8 T cells.

## Introduction

Viruses often exploit intracellular signaling pathways to facilitate entry, replication, latency, and reactivation. Among the many pathways that viruses manipulate is the phosphatidylinositol-3-kinase (PI3K)/Akt pathway. This pathway has several important activities including inhibiting apoptosis, regulating the cell cycle, and enhancing protein synthesis, resulting in increased cell survival and control of cell growth (1). Activation of the pathway by the binding of viruses, growth factors, or cytokines to receptors on the plasma membrane results in the movement of the PI3K complex from the cytoplasm to the plasma membrane. Class I PI3K complexes are important for virus infection and consist of a regulatory subunit (p50, p55, or p85) and a catalytic subunit (p110α, β, γ, or δ). The interaction of phosphorylated tyrosines on receptors with the p85 subunit of PI3K reduces its inhibitory effect on the p110 subunit, resulting in the phosphorylation of phosphatidylinositol 4, 5-triphosphate (PIP_2_) and the activation of downstream signaling molecules including PDK1, Akt, and mTOR. Mutations in the p85-regulatory subunit or the p110δ-catalytic subunit have been associated with immunodeficiencies often presenting with lymphoproliferative disease, recurrent respiratory infections, and severe herpesvirus infections (see below).

## Herpesviruses Modulate the PI3K Pathway

Eight herpesviruses infect humans: herpes simplex viruses (HSV) 1 and 2, varicella–zoster virus (VZV), cytomegalovirus (CMV), Epstein–Barr virus (EBV), human herpesviruses (HHV) 6 and 7, and Kaposi’s sarcoma-associated herpesvirus (KSHV). All herpesviruses infect and are shed from the epithelial cells, and all undergo latency and reactivation. HSV-1, HSV-2, and VZV establish latency in sensory neurons, CMV and HHV-6 in monocytes and CD34 cells, HHV-7 in CD4 cells, and EBV and KSHV in B cells. In healthy persons, HSV causes orolabial and genital herpes, VZV results in varicella and zoster, CMV, EBV, and HHV-6 cause mononucleosis, and HHV-6 and HHV-7 cause roseola. Each of the herpesviruses can result in a severe disease involving multiple organs in immunocompromised persons; CMV and EBV are frequently detected in the blood of immunocompromised persons. EBV is associated with lymphoproliferative disease in immunocompromised persons and B cell lymphoma, while KSHV is associated with Kaposi’s sarcoma and primary effusion lymphoma. While antibody contributes to protection from initial infection with herpesviruses, T cells are critical for reducing the severity of infection and disease associated with reactivation. Thus, mutations in genes important for the function of T cells can impair the control of herpesviruses by the host.

The PI3K pathway has a critical role for herpesvirus infection as well as for the control of herpesviruses by the immune system (2–4). Accordingly, herpesviruses manipulate this pathway to enhance virus entry, replication, latency, and reactivation. The binding of HSV (5), CMV (6), EBV (7), and KSHV (8) to the cell results in the activation of PI3K/Akt. Several viral glycoproteins including HSV gD and gB (9), CMV gB (10), EBV gp350 (7), and KSHV gB (11), each of which is required for initial infection of cells, activate the PI3K/Akt pathway. Viral proteins expressed during infection of cells by HSV (12), VZV (13), CMV (14), EBV (15), and KSHV (16) activate PI3K/Akt. These include the first proteins expressed in infected cells, the immediate-early proteins, including CMV IE1 and IE2 (14), EBV Rta (15) and KSHV Rta (17) which activate the PI3K/Akt pathway.

The PI3K/Akt pathway is also important for maintaining latency in HSV (18), EBV (19), and KSHV (20). Proteins and RNAs expressed during latency including HSV LAT (21) and EBV LMP1 (22), LMP2 (23), and EBNA2 (24) all activate the PI3K/Akt pathway. In addition, this pathway is critical for the reactivation of HSV (18), EBV (25), and KSHV (26) from latency.

Several herpesvirus proteins including HSV VP11 (27), VZV ORF12 (13), EBV LMP1 (22), and KSHV K1 (28) directly interact with the p85-regulatory subunit of PI3K to activate the PI3K/Akt pathway. Additional herpesvirus proteins interact with other proteins in the PI3K/Akt pathway.

## Immunodeficiencies Associated with Mutations in PIK3CD or PIK3R1

Two laboratories (29, 30) independently reported a new immunodeficiency due to heterozygous gain-of-function mutations in the p110δ-catalytic subunit of PI3K, which is encoded by *PIK3CD*. Angulo et al. (29) reported a series of 17 patients with activated PI3K-δ syndrome (APDS) due to an E1012K mutation in *PIK3CD*. Lymphocytes from the patients had increased levels of activated Akt and phosphatidylinositol 3, 4, 5-triphosphate (PIP_3_), and increased activation-induced cell death. The patients had lymphopenia, elevated levels of IgM and transitional B cells, and reduced levels of antibodies to *Streptococcus pneumoniae* and *Haemophilus influenzae* type B with a reduced number of circulating B cells and class-switched memory B cells. They also had repeated respiratory infections with damage to the lungs; some had severe virus infections. Lucas et al. (30) reported nine patients with p110δ-activating mutations causing senescent T cells, lymphadenopathy, and immunodeficiency (PASLI) with N334K, E525K, or E1012K mutations in *PIK3CD*. The patients’ lymphocytes had an increased phosphorylation of Akt and mTOR, an increased number of senescent effector T cells and transitional B cells, and a reduced number of naïve T cells, CD4 cells, and class-switched memory B cells. The patients had lymphoid hyperplasia often with obstructive lymphoid nodules and recurrent sinopulmonary infections; several patients had autoimmune cytopenias. Two had EBV lymphomas and all had EBV and/or CMV viremia.

Two laboratories (31, 32) reported a new immunodeficiency due to heterozygous gain-of-function mutations in the p85-regulatory subunit of PI3K, which is encoded by *PIK3R1*. These patients’ immune system and clinical phenotype were similar to those with *PIK3CD* mutations. Deau et al. (31) reported four patients with two different heterozygous splice mutations in *PIK3R1*, whose lymphocytes showed low numbers of memory B cells and naïve T cells, and increased levels of activated Akt, IgM, transitional B cells, senescent CD8 cells, and activation-induced cell death. One of the patients had CMV and EBV viremia and enterovirus enteritis. Lucas et al. (32) described four patients with heterozygous splice-site mutations in *PIK3R1*. Lymphocytes from the patients had an increased phosphorylation of Akt and an increased number of senescent effector T cells and CD8 cells; they had low numbers of CD4 cells and low levels of IgG. The patients had lymphoproliferative disease and frequent sinopulmonary infections.

With the recognition of similar phenotypes in patients with gain-of-function mutations in *PIK3CD* and *PIK3R1*, APDS and PASLI have now been divided into APDS1 and PASLI-CD when reporting patients with mutations in *PIK3CD*, and APDS2 and PASLI-R1 when reporting patients with mutations in *PIK3R1*. A different type of mutation was reported in *PIK3R1*. Conley et al. (33) reported a patient with a homozygous stop codon in the p85-regulatory subunit of PI3K. The patient had no B cells, normal numbers and activity of T cells, and no history of severe virus infection.

## Herpesvirus Infections in Patients with Gain-of-Function PIK3CD Mutations

The most frequent viral infectious complications associated with *PIK3CD* mutations have been EBV and CMV viremia and EBV lymphoproliferative disease or CMV lymphadenitis. In the first report of gain-of-function *PIK3CD* mutations (29), patients were screened for frequent respiratory infections and family histories of increased susceptibility to infection; accordingly, all 17 had recurrent upper or lower respiratory tract infections. Four of the 17 patients had infections caused by EBV, CMV, VZV, or HSV, including one patient with HSV pneumonia. In the next report of *PIK3CD* gain-of-function mutations (30), patients were screened based on the persistence of CMV and EBV in the peripheral blood; all nine patients had EBV viremia, with peak viral loads ranging from <250 to 63,350 copies/µl in the blood (30). Two patients had EBV-positive B cell lymphomas; one had an EBV-diffuse B cell lymphoma and one had EBV-nodular sclerosis Hodgkin disease. In one patient without lymphoma, two other family members had EBV lymphoma. Interestingly, while patients had high EBV DNA levels in the blood and some developed EBV lymphomas, in the patients who had EBV-specific CD8 T cells quantified by staining with HLA tetramers specific for EBV lytic and latency proteins, normal or elevated numbers of EBV-specific CD8 T cells were noted. Two patients had CMV viremia and three had CMV lymphadenitis. While it is possible that chronic infection with CMV or EBV could have resulted in the observed senescence of CD8 T cells, there was no correlation with the EBV and CMV load in the blood and the number of senescent CD8 T cells.

Since these two reports were published, additional papers have reported persistent herpesvirus viremia or severe herpesvirus infections in patients with *PIK3CD* gain-of-function mutations (Table 1). Mutations at seven sites in *PIK3CD*—E81K, G124D, N334K, C416R, E525K, E1021K, and E1025G—have been reported in persons with herpesvirus viremia or severe virus infections. In the largest review to date, a total of 53 patients with *PIK3CD* gain-of-function mutations were reported, and 49% had persistent or recurrent herpesvirus infections (40). EBV viremia was detected in 26% (14/53) of the patients, and 6% were reported to have a disseminated infection. EBV was detected in multiple biopsies including lymph nodes, tonsils, and the gastrointestinal tract as well as in the cerebrospinal fluid. Seven patients had diffuse lymphadenopathy; EBV and/or CMV was detected by PCR in the blood of six of these patients. One case of EBV encephalitis was reported. Two patients had EBV lymphomas, one had Hodgkin lymphoma and one had diffuse large B cell lymphoma; both patients died. CMV viremia was reported in 15% (8/53) of patients, 4 of whom responded to ganciclovir. EBV and CMV coinfection was reported in four patients, one of whom had a lymph node biopsy that was positive for EBV, CMV, and HHV-6 by PCR. Severe or persistent HSV or VZV infections were detected in 21% (11/53) of patients. One patient had HSV pneumonitis and one had recurrent HSV keratitis. Varicella infections resulted in hospitalization of two patients, and two had recurrent zoster.

**Table 1:** Viral infections in patients with germline gain-of-function mutations in *PIK3CD*.

Characteristic	Angulo et al. (29)	Lucas et al. (30)	Crank et al. (34)	Hartman et al. (35)	Kannan et al. (36)	Lawrence et al. (37)	Elgizouli et al. (38)	Dulau Florea et al. (39)^b^	Coulter et al. (40)^a^	Saerrini et al. (41)	Takeda et al. (42)	Chiriaco et al. (43)	Goto et al. (44)
Number of patients reported	17	9	3	5	1	1	5	10	53	1	3	1	1

Mutations	E1021K	N334K, E525K, E1021K	E1021K, C416R	E1021K	E1021K	NR	E1021K	E1021K, E525K, N334K, E1025G	E1021K, E525K	E1021K	G124D, E81K	E1021K	E1021K

EBV viremia	NR	9/9	1/3	NR	1/1	1/1	1/5	9/10	14/53	1/1	NR	1/1	1/1

EBV + lymphoma	NR	DLBCL in one, HL in one	NR	NR	NR	NR	NR	NR	DLBCL in one, HL in one	NR	NR	NR	NR

Other EBV disease	NR	NR	NR	NR	NR	NR	NR	NR	Encephalitis in one	NR	Two with lymphadenitis, one with fatal LPD	NR	lymphoid follicle in colon in one

CMV viremia	NR	2/8	NR	NR	0/1	1/1	1/5	4/10 viremia or lymphadenitis	8/53	NR	NR	NR	1/1

CMV lymphadenitis	NR	3/8	NR	NR	NR	1/1	NR	See above	NR	NR	NR	NR	NR

Other CMV diseases	NR	NR	NR	NR	NR	NR	One with systemic disease	NR	Four with systemic disease	NR	NR	NR	NR

Other severe herpesvirus infections	One with HSV pneumonia	NR	NR	One varicella pneumonia	NR	NR	NR	One with varicella after vaccine	Two with severe varicella, two with recurrent zoster, one with HSV keratitis, one with HSV pneumonitis	NR	Varicella after vaccine	NR	NR

Severe HPV infections	Severe warts in two patients	NR	NR	NR	NR	NR	NR	NR	Four with severe warts	NR	NR	NR	NR

Severe Molluscum contagiosum	NR	NR	NR	NR	NR	NR	NR	NR	Four with severe disease	NR	NR	NR	NR

Other viral infections	NR	NR	NR	NR	NR	NR	ADV viremia, norovirus GI disease for weeks	Two with ADV infection	Nine with ADV infection	NR	NR	NR	NR

CD4 cell numbers reduced	10/17	8/9	2/2	1/5	1/1	1/1	2/5	4/10	43/51	1/1	2/3	1/1	NR

CD8 cell numbers reduced	6/17	NR	0/1	NR	1/1	0/1	1/5	1/10	14/51	0/1	1/3	0/1	NR

NK cell numbers reduced	NR	2/9	0/1	NR	0/1	0/1	NR	NR	12/43	0/1	2/3	NR	NR

*^a^Twenty-five of the 53 patients were included in Angulo et al. (29)*.

*^b^Five of the 10 patients were included in Lucas et al. (30)*.

*EBV, Epstein–Barr virus; DLBCL, diffuse large B cell lymphoma; HL, Hodgkin lymphoma; LPD, lymphoproliferative disease; EBER, EBV-encoded RNA; CMV, cytomegalovirus; HSV, herpes simplex virus; HPV, human papillomavirus; ADV, adenovirus*.

Two other patients have been reported with *PIK3CD* gain-of-function mutations and EBV lymphomas (30). One patient had fatal EBV lymphoproliferative disease (42), two had EBV lymphadenitis (42), and one had EBV encephalitis (40). Two developed varicella after receiving the varicella vaccine (39, 42) and one had varicella pneumonia (35). Interestingly, despite frequent reports of CMV viremia and lymphadenitis, severe complications of CMV including pneumonia, colitis, or retinitis have not been reported. The treatment of patients with CMV lymphadenitis is often unsatisfactory; while the disease responds to antiviral therapy, it often recurs when treatment is stopped.

## Herpesvirus Infections in Patients with Gain-of-Function PIK3R1 Mutations

In the first report of patients with gain-of-function *PIK3R1* mutations, Deau et al. (31) described four patients with recurrent respiratory bacterial infections, two of whom had EBV viremia and one of whom had both CMV and EBV viremia (9,300 and 1,500 copies/ml, respectively). In the next report of *PIK3R1* gain-of-function mutations, Lucas et al. (32) reported four patients, one of whom had CMV lymphadenitis. Since these two papers were published, additional papers have reported persistent EBV or CMV viremia or severe herpesvirus infections in patients with gain-of-function mutations in *PIK3R1* (Table 2). All patients with severe virus infections have had splice donor-site mutations resulting in loss of exon 11. In the largest series to date, Elkaim et al. (45) reported 36 patients with mutations in *PIK3R1*. EBV viremia was detected in 22% (8/36) of patients, 4 of whom were asymptomatic and 4 of whom had EBV lymphoproliferative disease. One of these patients had two episodes of EBV Hodgkin lymphoma. Asymptomatic CMV viremia was present in 17% (6/36) of patients, and two had CMV lymphadenitis. Two patients were hospitalized for severe VZV infections. In another report (47), a 15-year-old boy had CMV viremia and CMV lymphadenitis that was refractory to therapy. A lymph node biopsy showed 240,000 copies of CMV/mg of tissue and follicular hyperplasia. While he initially responded to ganciclovir and valganciclovir, his lymphadenopathy recurred associated with the obstruction of the upper airway. A repeat lymph node biopsy was CMV-positive, and he received additional valganciclovir and corticosteroids and had a good response, though he had intermittent low-grade CMV viremia. He later presented with recurrent massive lymphadenopathy, and a repeat lymph node biopsy was CMV-positive, and he was treated again with ganciclovir and valganciclovir. He relapsed once valganciclovir was stopped and when he became refractory to antivirals, hematopoietic stem cell transplantation was performed, and he responded well. Like patients with mutations in *PIK3CD*, no cases of severe CMV involving the lungs, colon, liver, or retina have been reported in patients with mutations in *PIK3R1*.

**Table 2:** Viral infections in patients with germline gain-of-function mutations in PIK3R1.

Characteristic	Deau et al. (31)	Lucas et al. (32)	Elkaim et al. (45)	Olbrich et al. (46)	Kuhlen et al. (47)	Bravo Garcia-Morato et al. (48)	Hauck et al. (49)
Number of patients reported	4	4	36^a^	2	1	2	3

Mutation	Splice donor-site mutations resulting in loss of exon 11	Splice donor-site mutations resulting in loss of exon 11	Splice donor-site mutations resulting in loss of exon 11	Splice donor-site mutations resulting in loss of exon 11	Splice donor-site mutations resulting in loss of exon 11	Splice donor-site mutations resulting in loss of exon 11	Splice donor-site mutations resulting in loss of exon 11

EBV viremia	1/4	0/3	8/36	0/2	0/1	1/2	0/3

EBV + lymphoma	NR	0/3	One with HL	NR	NR	NR	1 DBCL

Other EBV diseases	NR	0/3	Four with EBV LPD	NR	NR	NR	NR

CMV viremia	1/4	0/3	6/35	2/2	1/1	0/2	NR

CMV lymphadenitis	NR	1/3	2	NR	1/1	NR	NR

Other severe herpesviruses	NR	NR	Two hospitalized for varicella	NR	NR	NR	NR

Other severe viral infections	Enterovirus enteritis	NR	One with measles encephalitis, two with chronic HBV, one with chronic HCV	ICU hospitalization for RSV	NR	NR	NR

CD4 cell numbers reduced	1/4	NR	8/23	0/2	NR	1/2	1/3

CD8 cell numbers reduced	0/4	NR	1/23	0/2	NR	0/2	0/3

NK cell numbers reduced	1/4	NR	NR	NR	NR	0/2	0/3

*^a^Eight of the 36 were reported in Deau et al. (31) or Lucas et al. (32)*.

*EBV, Epstein–Barr virus; HL, Hodgkin lymphoma; DBCL, diffuse large B cell lymphoma; LPD, lymphoproliferative disease; CMV, cytomegalovirus; HBV, hepatitis B virus; HCV, hepatitis C virus; ICU, intensive care unit; RSV, respiratory syncytial virus*.

While most case reports of patients with EBV or CMV viremia did not indicate the level of viral DNA, in 13 patients with EBV viremia and 5 with CMV viremia and mutations in either *PIK3CD* or *PI3KR1*, the levels were quantified and expressed as copies of viral DNA/ml. In these cases, the mean and median EBV loads were 9,146 and 2,250 copies/ml, respectively, and the mean and median CMV loads were 2,749 and 1,211 copies/ml, respectively. Thus, the levels of EBV and CMV in the blood generally were not markedly elevated. EBV and CMV viremia were not reported as initiating with symptomatic primary infection; instead, they were associated with virus reactivation.

## Other Virus Infections in Patients with Gain-of-Function PIK3CD Mutations

In the first report of gain-of-function *PIK3CD* mutations, two patients were described with severe warts (29); subsequently, two additional patients with severe warts have been reported (40). Four patients have been reported with severe Molluscum contagiosum infections (40), one with adenovirus viremia (38), and 11 others reported with adenovirus infections with virus isolated from the blood, bronchoalveolar lavage fluid, and/or stool (39, 40). One patient was reported with norovirus infection that lasted for several weeks and was associated with persistent diarrhea (38). CD4 T cell numbers were reduced in 72% of patients, and CD8 T cell and NK cell numbers were reduced in 27% of patients with severe virus infections and *PIK3CD* mutations (Table 1).

## Other Virus Infections in Patients with Gain-of-Function PIK3R1 Mutations

In the first report of gain-of-function *PIK3R1* mutations, one patient was reported with enterovirus gastroenteritis (31). In the largest report of *PIK3R1* mutations to date, Elkaim et al. (45) reported one patient with measles encephalitis and hydrocephalus and other patients with chronic hepatitis B and hepatitis C virus infections. In another report, one patient was hospitalized in the intensive care unit for bronchiolitis due to respiratory syncytial virus infection (46). CD4 T cell numbers were reduced in 32% of patients and NK cell numbers were reduced in 11% of patients with severe virus infections and *PIK3R1* mutations (Table 2).

## Mechanism for Impaired Control of Herpesvirus Infections in Patients with PI3K Mutations

Lucas et al. (30) found that patients with *PIK3CD* gain-of-function mutations had normal or high levels of EBV-specific CD8 T cells in the blood by tetramer staining, but that EBV-specific CD8 T cells were predominantly CCR7^−^CD45RA^−^ indicative of terminal effector memory cells and had more CD38 than control cells indicative of an increased activity. They postulated that the persistent hyperactivation of Akt results in an increase in the proliferation of CD8 T cells and an increase in the number of terminal differentiated effector CD8 T cells with a corresponding increase in senescent CD8 T cells and a decrease in long-lived memory CD8 T cells. Together, this may result in impaired control of EBV- and CMV-infected cells. The increased proliferation of EBV-infected B cells could also increase the risk for additional chromosomal mutations and result in an increased risk of EBV lymphomas. Interestingly, while older persons have a similar number of T cells than younger persons, they have a higher frequency of senescent T cells (50, 51). Older persons also have higher levels of EBV and CMV in the blood than younger persons (52, 53), and older persons may develop EBV lymphoproliferative disorders in the absence of an immune deficiency disease (54). Thus, an increased number of senescent T cells has been associated with impaired control of EBV and CMV infections. Coulter et al. (40) also noted that a reduced number of CD4, CD8, or NK cells was not associated with herpesvirus infections in patients with *PIK3CD* mutations, indicating that a functional rather than a quantitative abnormality in lymphocytes was responsible for the infections.

Cytomegalovirus and EBV persist predominantly in the blood, lymph nodes, and spleen, and the disease is often associated with lymphadenopathy, lymphadenitis, or lymphomas involving the lymphoid tissues. By contrast, HSV and VZV are latent in the nervous system and most often result in disease involving the skin. CD8-naïve and central or effector memory T cells are the predominant T cell types in the blood, spleen, and lymph nodes, while tissue-resident memory T cells and terminally differentiated effector CD8 T cells are the predominant CD8 T cell subsets in the peripheral tissues including the skin (55, 56). In addition, the persistence of EBV and CMV in the blood allows for clonal expansions of T cells, with the persistence of memory T cells after initial infection. Thus, the increase in terminal differentiated effector CD8 T cells and the reduction in memory CD8 T cells in patients with mutations in *PIK3CD* or *PIK3R1* may allow EBV and CMV to proliferate in the blood and lymphoid organs while having less of an effect on HSV and VZV in the skin. Furthermore, the reduction in memory CD8 T cells in the blood and lymphoid tissues may allow EBV and CMV to proliferate to higher levels resulting in viremia, lymphadenitis, and EBV lymphoma. In addition, the increased numbers of terminal differentiated effector CD8 T cells in patients with *PIK3CD* or *PIK3R1*, which are generally more often present in the peripheral tissues than in the blood, may have a protective effect from CMV involvement of the lungs, colon, and liver in these patients.

## Screening and Treatment

Since EBV viremia was detected in 46 and 22% of patients with mutations in *PIK3CD* and *PIK3R1*, respectively, and CMV viremia was detected in 20 and 21% of patients with mutations in *PIK3CD* and *PIK3R1*, respectively, testing for mutations in these two genes should be considered in patients with unexplained EBV or CMV viremia. The frequency of other virus infections in these patients is much lower, and therefore screening for mutations in *PIK3CD* or *PIK3R1* would be less likely to be useful.

The treatment of patients with *PIK3CD* mutations with a PI3K inhibitor, leniolisib (57), or with an mTOR inhibitor, rapamycin (30, 40) reduced lymphoproliferation and the number of senescent T cells and increased the number of naïve T cells. Despite its immunosuppressive effects, patients with *PIK3CD* mutations treated with leniolisib did not have an increase in EBV or CMV viremia while on therapy (57). Similarly, complications associated with EBV or CMV have not been reported in patients treated with rapamycin (30, 40). Rapamycin has been shown to inhibit the development of EBV-positive B cell lymphomas in a mouse model (58) and has been associated with the resolution of EBV-positive lymphoproliferative disease in patients (59). Similarly, rapamycin modestly reduces CMV replication *in vitro* (60), and the use of rapamycin instead of other immunosuppressant drugs has resulted in the reduction in CMV infection and disease (61). Thus, despite its immunosuppressive activities, rapamycin or PI3K inhibitors do not appear to increase the risk of EBV or CMV disease in patients with *PIK3CD* mutations.

## Author Contributions

The author confirms being the sole contributor of this work and approved it for publication.

## Conflict of Interest Statement

The author declares that the research was conducted in the absence of any commercial or financial relationships that could be construed as a potential conflict of interest.
